# Editorial: Neural Substrates of Acupuncture: From Peripheral to Central Nervous System Mechanisms

**DOI:** 10.3389/fnins.2019.01419

**Published:** 2020-01-17

**Authors:** Vitaly Napadow, Florian Beissner, Yiwen Lin, Younbyoung Chae, Richard E. Harris

**Affiliations:** ^1^Martinos Center for Biomedical Imaging, Massachusetts General Hospital, Harvard Medical School, Boston, MA, United States; ^2^Somatosensory and Autonomic Therapy Research, Hannover Medical School, Hanover, Germany; ^3^Graduate Institute of Acupuncture Science, College of Chinese Medicine, China Medical University, Taichung, Taiwan; ^4^Acupuncture and Meridian Science Research Center, Kyung Hee University, Seoul, South Korea; ^5^Department of Anesthesiology, Chronic Pain and Fatigue Research Center, University of Michigan, Ann Arbor, MI, United States

**Keywords:** acupuncture, neuroscience, somatosensation, endorphin, autonomic nervous system

“Acupuncture” is a therapeutic intervention involving percutaneous mechanical, thermal, or electrical stimulation via needling of specific locations on the body. Acupuncture has been used in China and other Asian countries for thousands of years, and is one component of traditional Chinese medicine. Usage of acupuncture in Western countries, such as the United States, has been growing slowly but steadily (Ma et al., 2016; Nahin et al., 2016), with most common applications for chronic pain and neurological conditions. Mechanistic research exploring how acupuncture may reduce pain and contribute to other clinically meaningful outcomes is ongoing. This special issue further explores the neural basis of acupuncture, focusing on both peripheral and central nervous system mechanisms.

Historically, in the West, acupuncture research began mainly in the 1970's. An early popular theory to explain acupuncture analgesia was the “gate control theory,” which posited that stimulation from acupuncture needling produces pre-synaptic inhibition of nociceptive afference in the central nervous system (Melzack and Wall, 1965). However, the gate control theory would predict analgesia on the order of milliseconds, while acupuncture analgesia develops much more slowly, reaching a maximum analgesic effect 30 min after initial stimulation (Pomeranz, 1989). Such results seemingly refuted the previously popular gate control theory. However, other research studies in the 1970's clearly linked peripheral and central nervous system signaling in acupuncture mechanisms of action for pain relief. Naloxone, an opioid receptor antagonist, has been shown to block acupuncture analgesia by binding to selective opioid receptors (Mayer et al., 1977). In fact, acupuncture analgesia has been demonstrated to be mediated by afferent sensory nerves, as vascular occlusion has no effect on acupuncture analgesia (Chiang et al., 1973), while procaine (a local anesthetic) has indeed been shown to negate acupuncture analgesia (Ulett et al., 1998).

These early studies have been bolstered by more recent human and pre-clinical basic science studies, further defining the mechanistic role of endogenous opioid receptors in acupuncture analgesia (Harris et al., 2009), and suggesting other mechanisms of action including adenosine receptor modulation (Goldman et al., 2010), dopamine signaling for immune regulation (Torres-Rosas et al., 2014), and neuroplasticity in the primary somatosensory cortex of the brain (Maeda et al., 2017). Indeed, clinical trials of acupuncture for chronic pain conditions indicate that acupuncture analgesic effects persist for months following the end of treatment suggesting long term plastic changes in pain processing (MacPherson et al., 2017). This type of long term change in sensory processing is a hallmark of central neurobiology. These studies over the last 50 years of acupuncture research clearly highlight the nervous system as the main conduit for clinical efficacy underlying this therapeutic intervention.

This Issue furthers the research base on the neural basis of acupuncture mechanisms. Studies include both behavioral and neuroimaging research in humans, as well as basic molecular, cellular, and physiological research in animal models. Clinical applications included in this Issue extend beyond pain, to cover chronic itch, depression, stroke, drug addiction, and cardiovascular regulation. Molecular and cellular targets are identified for acupuncture analgesia and anti-depressive effects (Wan et al.; Zheng et al.), as well as inflammatory modulation (Ma et al.) and characterization of specific body locations (i.e., acupoints) for needle stimulation (Fan et al.). Other preclinical studies highlight specific brain regions such as paraventricular nucleus for cardiovascular modulation by acupuncture (Cheng et al.) and cuneate nucleus in drug dependence (Chang et al.). Human research studies explore the role of the brain's default mode network, previously implicated in acupuncture-evoked brain response and analgesia (Hui et al., 2009; Napadow et al., 2012), in acupuncture clinical response more broadly (Zhang et al.). Human neuroimaging studies in this issue also evaluate potential brain-based mechanisms of acupuncture for motor outcomes (Nierhaus et al.) and itch (Min et al.). Human behavioral studies evaluate acupuncture modulation of temporal summation and its role in analgesia (Baeumler et al.). The role of cognitive effects and expectancy, vis-à-vis the placebo effect, are also investigated as they relate to acupuncture (Aichner et al.; Lee et al.; Musial; Song et al.). Other studies evaluate neural modulation by combined acupuncture with mental imagery (Takahashi et al.) or transcranial magnetic stimulation (Huang et al.), and the more general role of acupuncture in stress reduction (Lee et al.). Finally, structural parameters of the needle are evaluated for their role in analgesia (Bae et al.) and an interesting study explores how the connective tissue system and nervous system may be differentially involved in acupuncture efficacy (Chang et al.).

We hope the reader enjoys this compendium of novel acupuncture research studies, which both extends the substantive research base supporting acupuncture mechanisms of action, and bridges to future basic and translational research that more precisely defines the pathways and neural circuitry underpinning acupuncture clinical efficacy.

## Author Contributions

All authors listed have made a substantial, direct and intellectual contribution to the work, and approved it for publication.

### Conflict of Interest

The authors declare that the research was conducted in the absence of any commercial or financial relationships that could be construed as a potential conflict of interest.
